# Supplementary material for: A Flagellin-Adjuvanted Trivalent Mucosal Vaccine Targeting Key Periodontopathic Bacteria
Source: Vaccines (Basel). 2024 Jul 8;12(7):754. doi: 10.3390/vaccines12070754 (PMC11281409; doi:10.3390/vaccines12070754)
Supplement: Supplementary file 1 [file vaccines-12-00754-s001.zip › vaccines-3059013-supplementary figures.pdf]

Figure S1

a

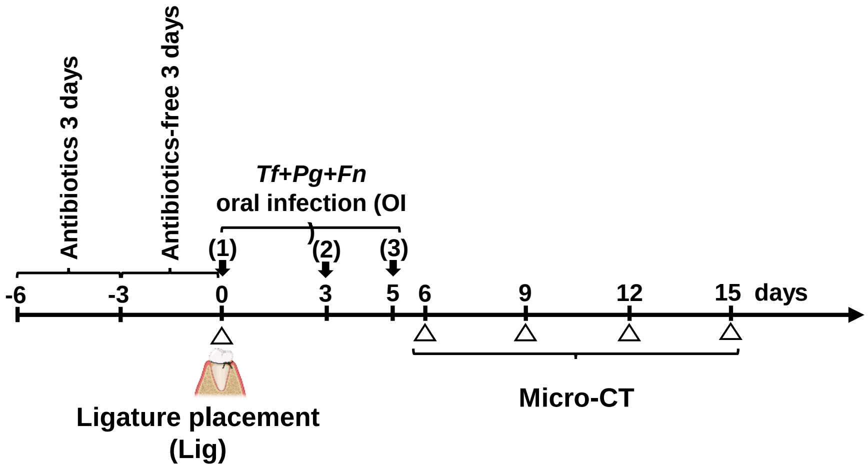

Antibiotics 3 days
Antibiotics-free 3 days
Tf+Pg+Fn
oral infection (OI)
(1)
(2)
(3)
-6
-3
0
3
5
6
9
12
15
days
Ligature placement
(Lig)
Micro-CT

b

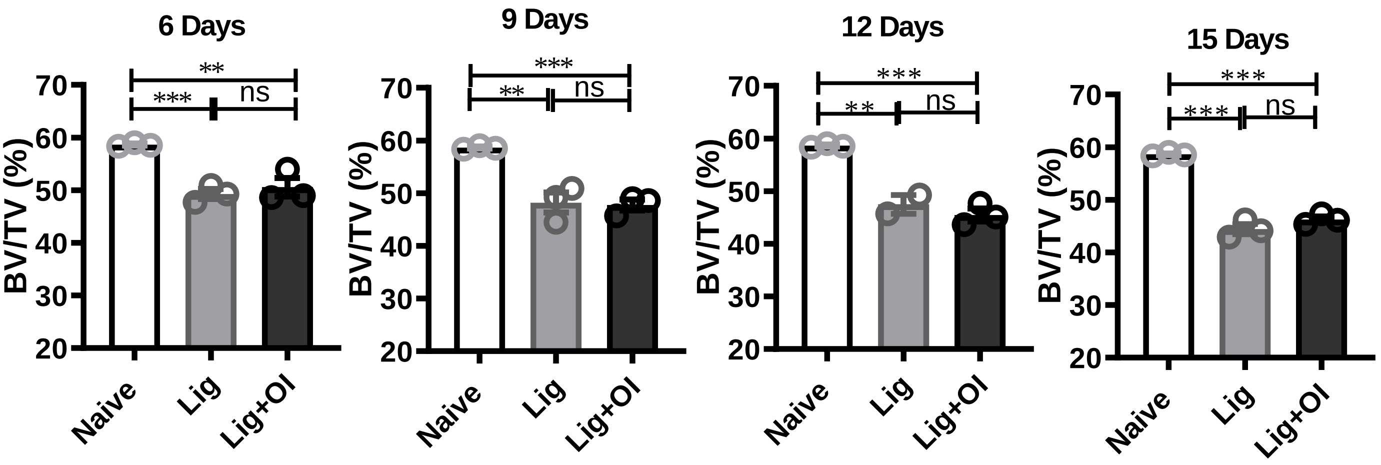

6 Days
9 Days
12 Days
15 Days
BV/TV (%)
70
60
50
40
30
20
**
***
ns
Naive
Lig
Lig+OI

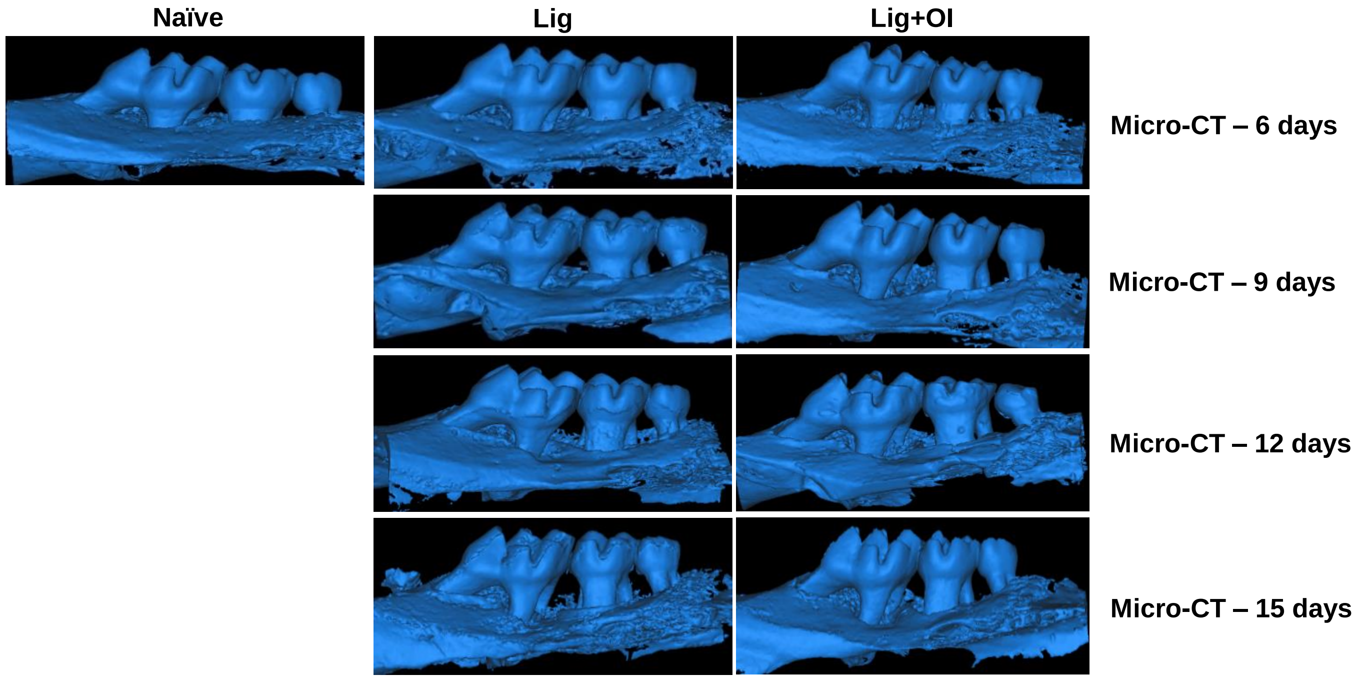

Naïve
Lig
Lig+OI
Micro-CT – 6 days
Micro-CT – 9 days
Micro-CT – 12 days
Micro-CT – 15 days

Figure S2

a

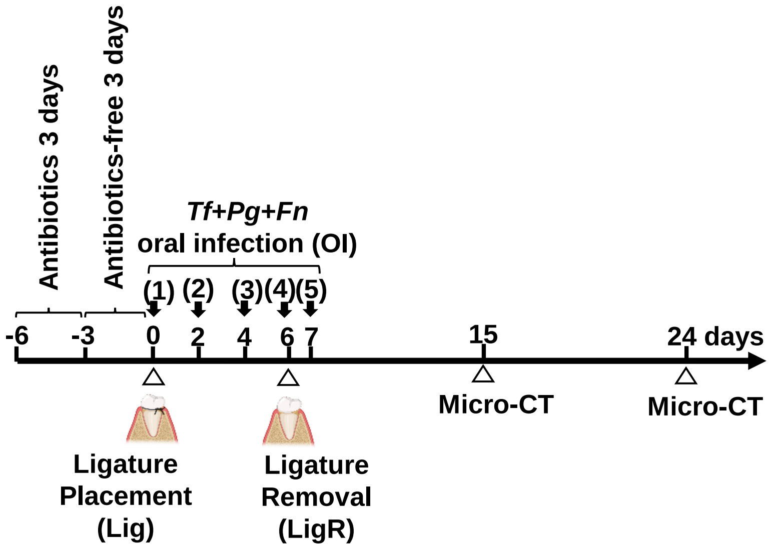

b

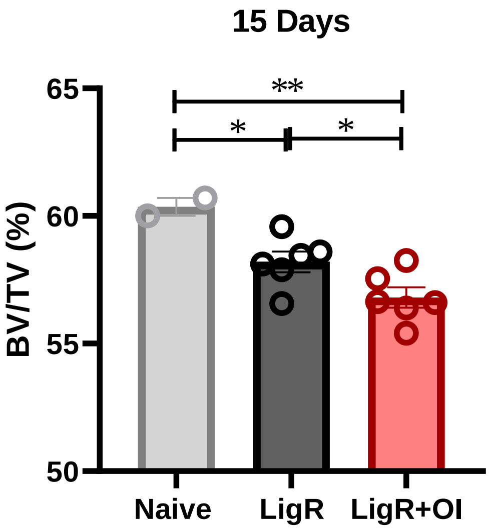

C

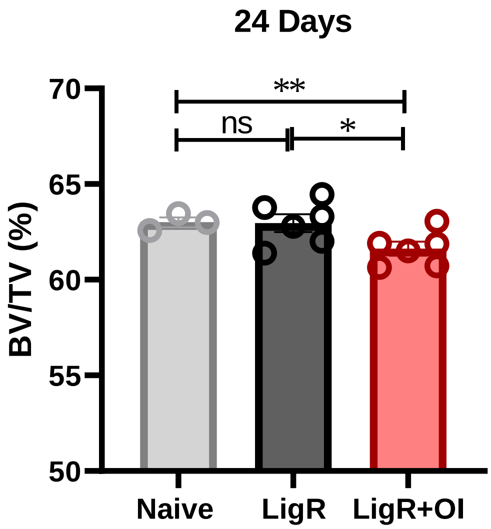

Figure S3

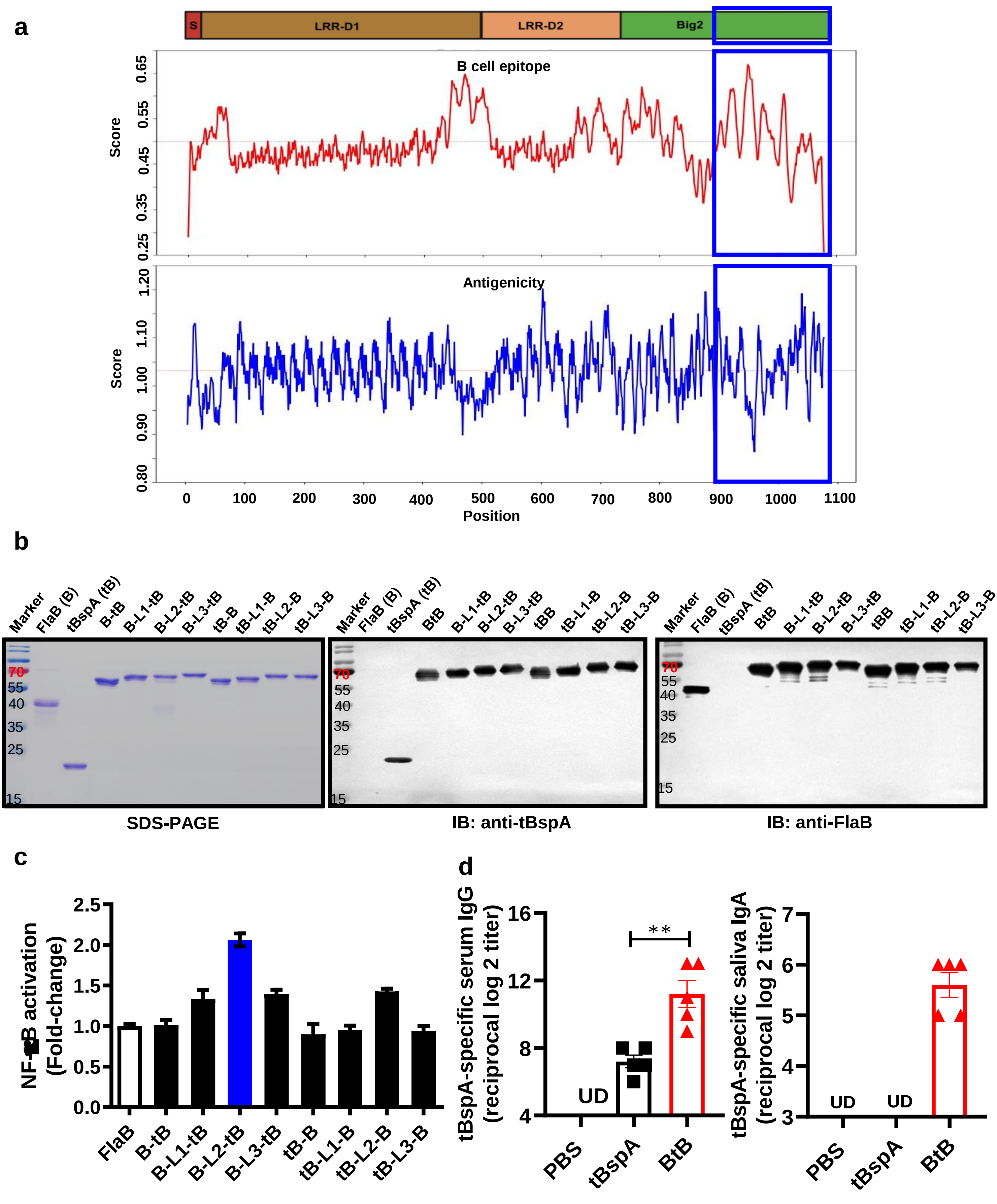
a
S
LRR-D1
LRR-D2
Big2
B cell epitope
Score
Antigenicity
Position
b
Marker
FlaB (B)
tBspA (tB)
B-tB
B-L1-tB
B-L2-tB
B-L3-tB
tB-B
tB-L1-B
tB-L2-B
tB-L3-B
SDS-PAGE
IB: anti-tBspA
IB: anti-FlaB
c
NF-κB activation (Fold-change)
FlaB
B-tB
B-L1-tB
B-L2-tB
B-L3-tB
tB-B
tB-L1-B
tB-L2-B
tB-L3-B
d
tBspA-specific serum IgG (reciprocal log 2 titer)
tBspA-specific saliva IgA (reciprocal log 2 titer)
UD
**
PBS
tBspA
BtB

Figure S4

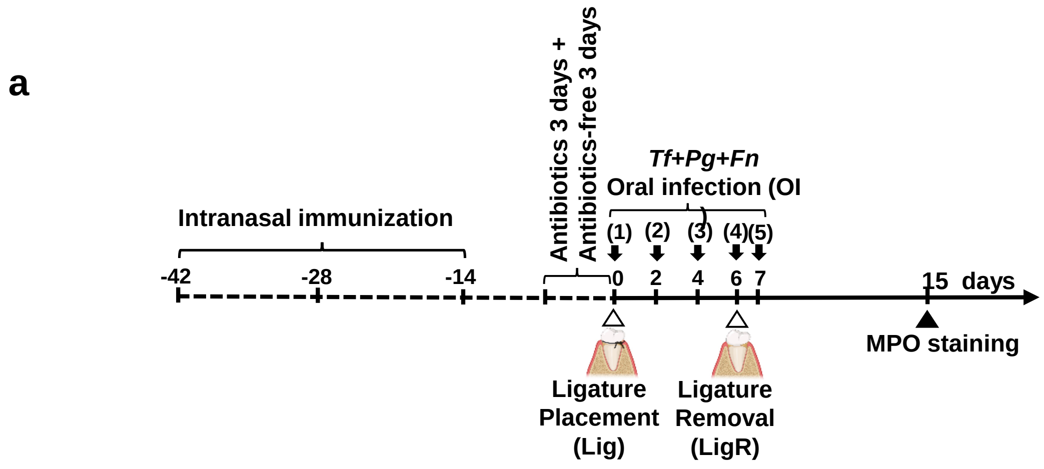

a
Intranasal immunization
Antibiotics 3 days +
Antibiotics-free 3 days
Tf+Pg+Fn
Oral infection (OI)
(1) (2) (3) (4)(5)
-42
-28
-14
0
2
4
6
7
15 days
MPO staining
Ligature
Placement
(Lig)
Ligature
Removal
(LigR)

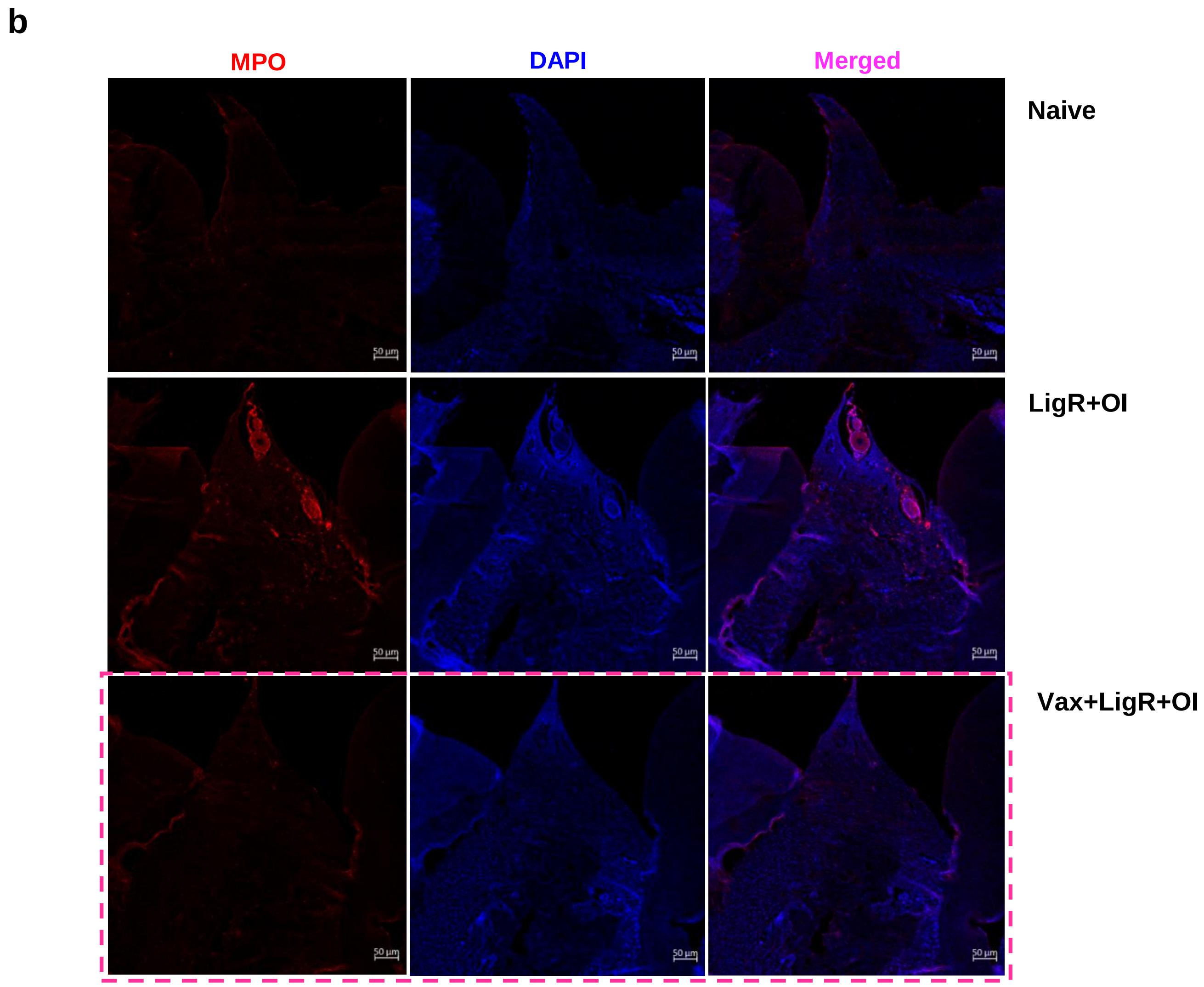

b
MPO
DAPI
Merged
Naive
LigR+OI
Vax+LigR+OI

Figure S5

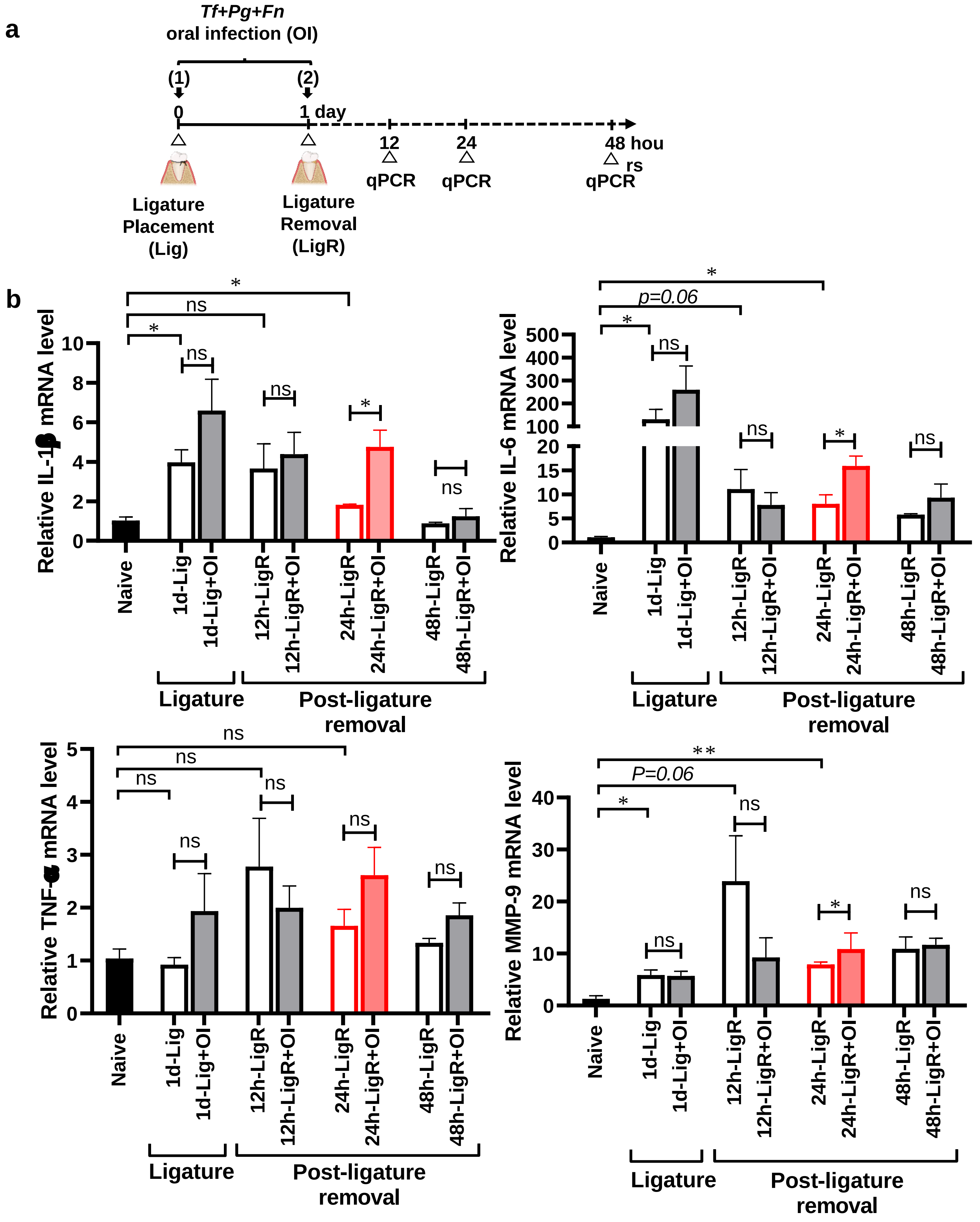
a
Tf+Pg+Fn
oral infection (OI)
(1)
(2)
0
1 day
12
24
48 hou rs
qPCR
qPCR
qPCR
Ligature Placement (Lig)
Ligature Removal (LigR)
b
Relative IL-1β mRNA level
Relative IL-6 mRNA level
Relative TNF-α mRNA level
Relative MMP-9 mRNA level
p=0.06
P=0.06
ns
*
**
Naive
1d-Lig
1d-Lig+OI
12h-LigR
12h-LigR+OI
24h-LigR
24h-LigR+OI
48h-LigR
48h-LigR+OI
Ligature
Post-ligature removal

Figure S6

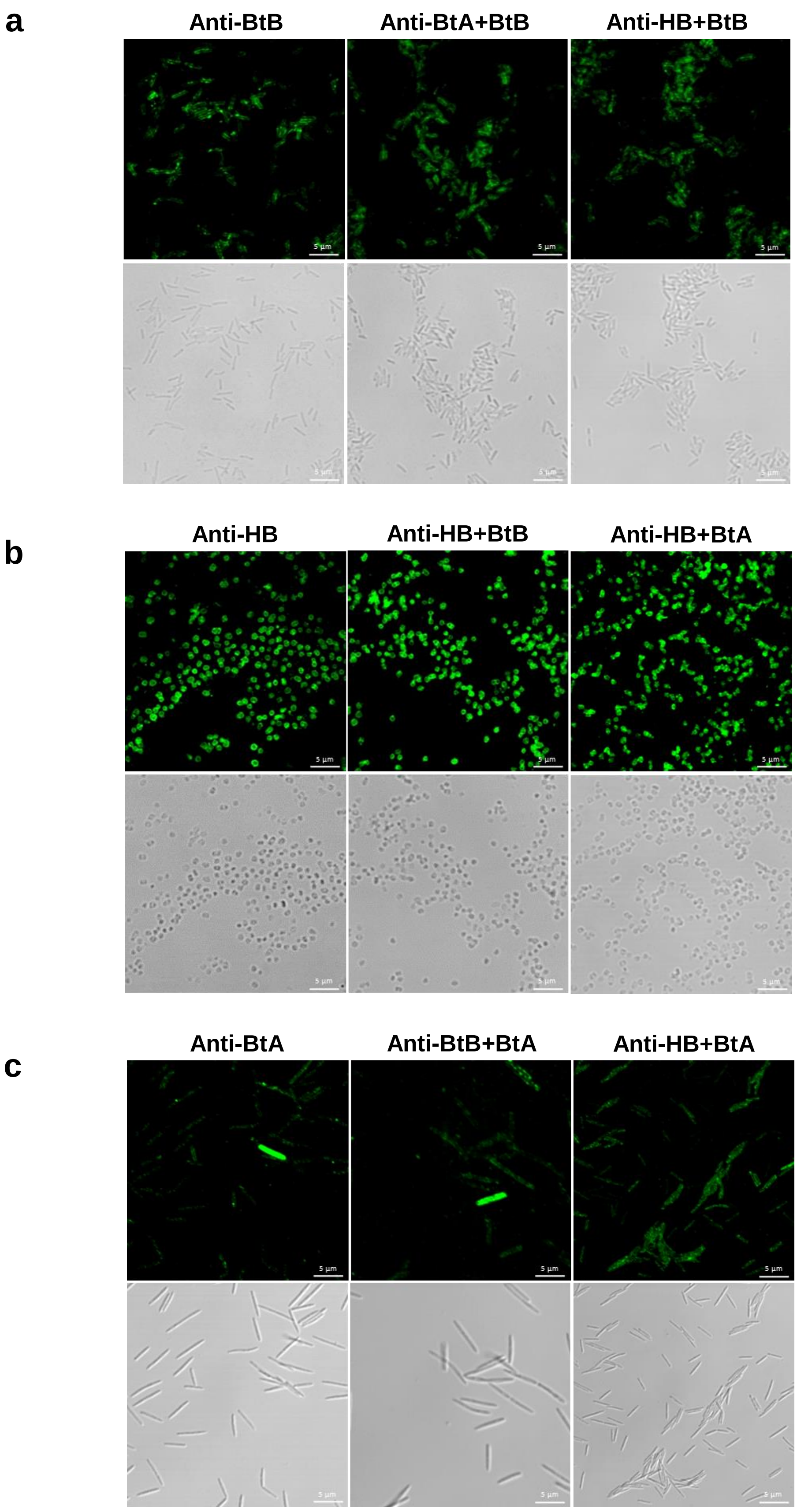

a
Anti-BtB
Anti-BtA+BtB
Anti-HB+BtB
b
Anti-HB
Anti-HB+BtB
Anti-HB+BtA
c
Anti-BtA
Anti-BtB+BtA
Anti-HB+BtA

Figure S7

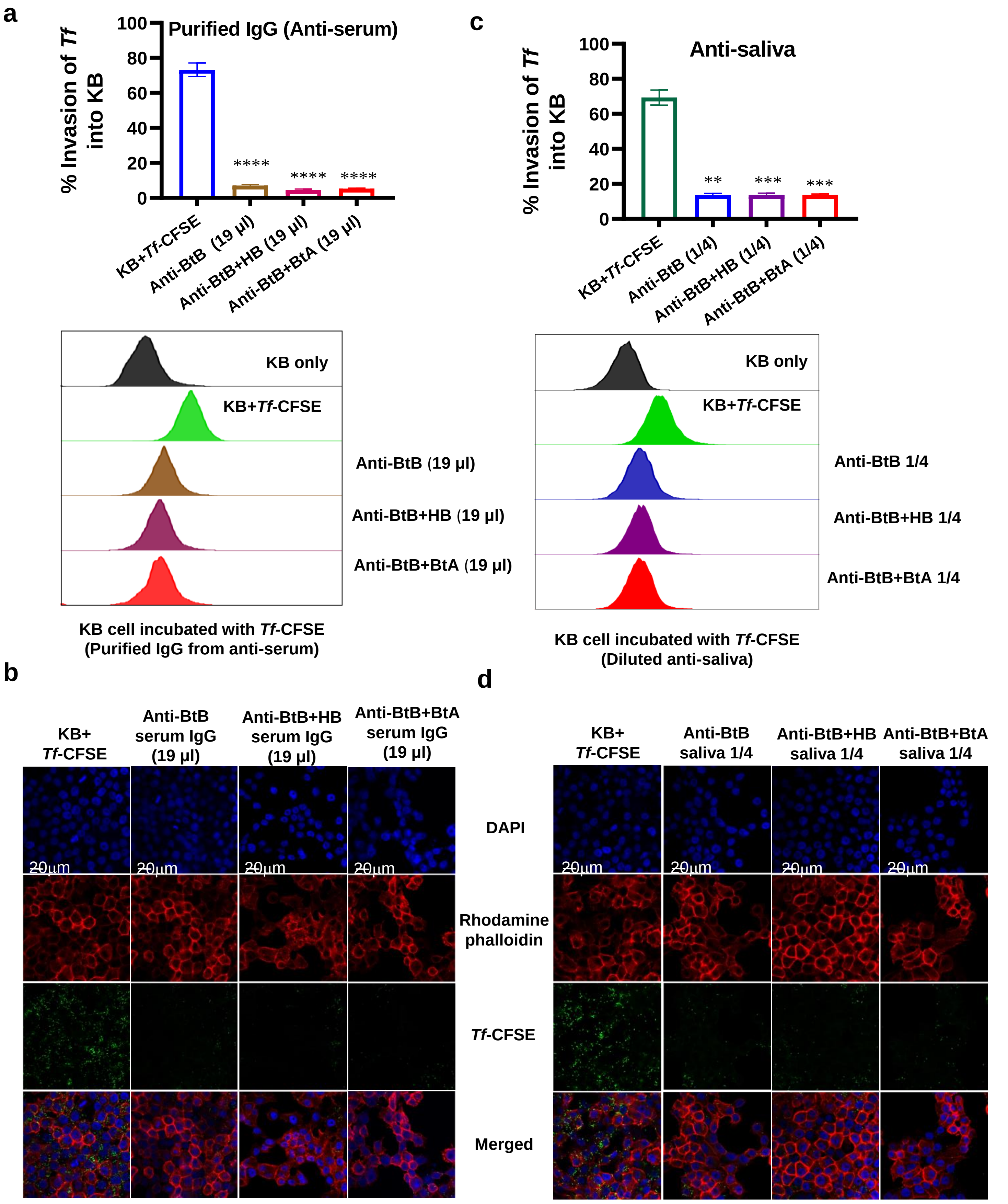
a
Purified IgG (Anti-serum)
% Invasion of Tf into KB
100
80
60
40
20
0
****
****
****
KB+Tf-CFSE
Anti-BtB (19 µl)
Anti-BtB+HB (19 µl)
Anti-BtB+BtA (19 µl)
KB only
KB+Tf-CFSE
Anti-BtB (19 µl)
Anti-BtB+HB (19 µl)
Anti-BtB+BtA (19 µl)
KB cell incubated with Tf-CFSE
(Purified IgG from anti-serum)
c
Anti-saliva
% Invasion of Tf into KB
100
80
60
40
20
0
**
***
***
KB+Tf-CFSE
Anti-BtB (1/4)
Anti-BtB+HB (1/4)
Anti-BtB+BtA (1/4)
KB only
KB+Tf-CFSE
Anti-BtB 1/4
Anti-BtB+HB 1/4
Anti-BtB+BtA 1/4
KB cell incubated with Tf-CFSE
(Diluted anti-saliva)
b
KB+ Tf-CFSE
Anti-BtB serum IgG (19 µl)
Anti-BtB+HB serum IgG (19 µl)
Anti-BtB+BtA serum IgG (19 µl)
20µm
DAPI
Rhodamine phalloidin
Tf-CFSE
Merged
d
KB+ Tf-CFSE
Anti-BtB saliva 1/4
Anti-BtB+HB saliva 1/4
Anti-BtB+BtA saliva 1/4

Figure S8

a

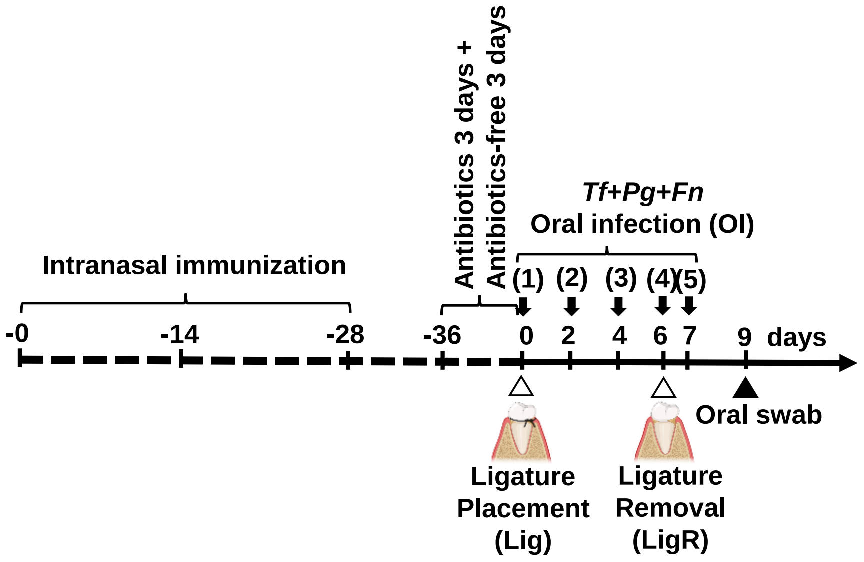

Antibiotics 3 days +
Antibiotics-free 3 days
Tf+Pg+Fn
Oral infection (OI)
Intranasal immunization
(1) (2) (3) (4) (5)
-0
-14
-28
-36
0
2
4
6
7
9
days
Oral swab
Ligature
Placement
(Lig)
Ligature
Removal
(LigR)

b

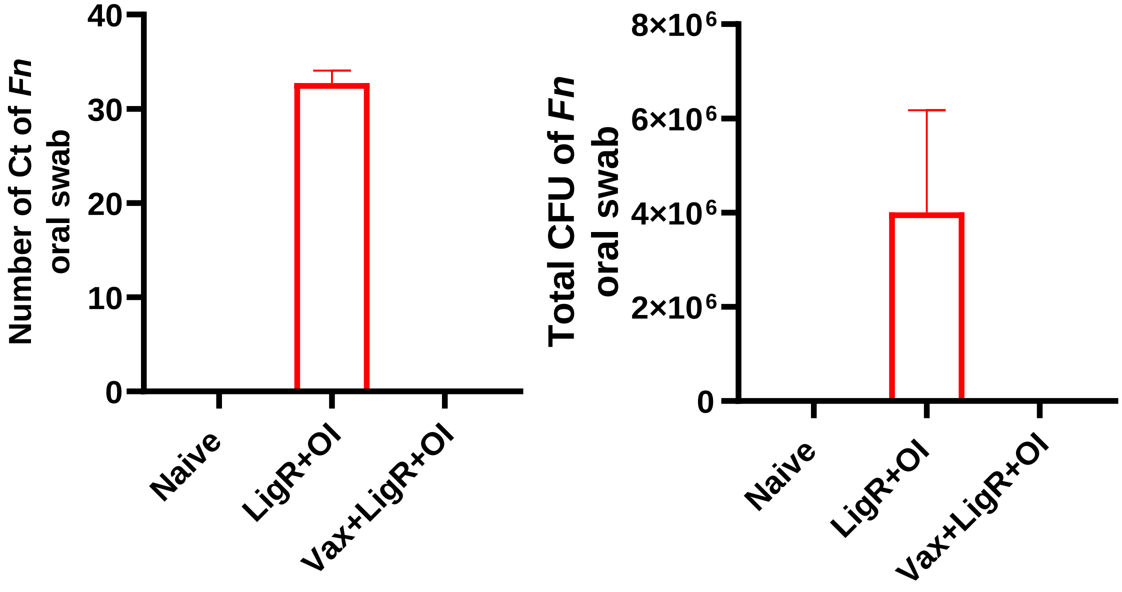

Number of Ct of Fn
oral swab
40
30
20
10
0
Naive
LigR+OI
Vax+LigR+OI
Total CFU of Fn
oral swab
8×10^6
6×10^6
4×10^6
2×10^6
0
Naive
LigR+OI
Vax+LigR+OI
