# Supplementary material for: A Flagellin-Adjuvanted Trivalent Mucosal Vaccine Targeting Key Periodontopathic Bacteria
Source: Vaccines (Basel). 2024 Jul 8;12(7):754. doi: 10.3390/vaccines12070754 (PMC11281409; doi:10.3390/vaccines12070754)
Supplement: Supplementary file 1 [file vaccines-12-00754-s001.zip › vaccines-3059013-supplementary.pdf]

Flagellin-adjuvanted trivalent mucosal vaccine targeting key periodontopathic bacteria

Vandara Loeurng, Sao Puth, Seol Hee Hong, Yun Suhk Lee, Kamalakannan Radhakrishnang, Jeong Tae Koh, Joong-Ki Kook, Joon Haeng Rhee, Shee Eun Lee

Supplementary Information

Supplementary figure legends

Supplementary Figure S1. A ligature-induced periodontitis with mixed oral bacterial infection of *Tf*, *Pg*, and *Fn* model. (**a**) Schedule of the experimental approach combining ligature-induced periodontitis with oral infection using a mixture of *Tf*, *Pg*, and *Fn* in BALB/c mice. Ten-week-old female BALB/c mice were subjected to ligature-induced periodontitis by placing silk around the second maxilla (M2) and orally infected with a mixture of *Tf*, *Pg*, and *Fn* three times at days 0, 3, and 5. (**b**) Micro-CT analysis for alveolar bone loss. Bone volume density (BV/TV) of alveolar bone resorption in mice was measured at various time points (6, 9, 12, and 15 days) post-ligature placement (upper panels). Representative 3D image of maxillary palatal side bone destruction (lower panels). Naïve, control mice; Lig, mice with ligature placement; Lig+OI, mice with ligature placement plus oral bacterial infections. The data are presented as the mean ± SEM in each group. $n \leq 3$, $^{**}P < 0.01$, $^{***}P < 0.001$, NS non-significant.

Supplementary Figure S2. Establishment of a temporary ligature plus oral infection model. (**a**) An experimental schedule of modifying ligature-induced periodontitis with oral infection of *Tf*, *Pg*, and *Fn* in BALB/c mice. The ligature was applied on day 0, followed by 3 times oral bacterial infections with mixed suspension of *Tf*, *Pg*, and *Fn* at 2-day intervals. On the 6th day post-ligature placement, the ligature was removed, and 2 additional rounds of oral bacterial infection. Alveolar bone resorption levels BV/TV were measured at 8 days (day 15) (**b**) and at 17 days (day 24) (**c**) after the last infection. Naïve, control mice; LigR, mice with ligature placement/removal; LigR+OI, mice with ligature placement/removal plus mixed oral bacterial infection. The data are presented as the mean ± SEM in each group. $n \leq 6$; $^{*}P < 0.05$; $^{**}P < 0.01$; NS non-significant

Supplementary Figure S3. Development and characterization of FlaB-L2-tBspA (BtB) targeting *Tf.* **(a)** Selection of truncated BspA (tBspA) as vaccine antigen. Computational analysis of the BspA domain selected truncated BspA (tBspA) as a vaccine antigen against *Tf.* The optimal tBspA, indicated in the blue box region, was chosen through a computational approach involving BepiPred 2.0 (B cell epitope) and Tongaokar antigenicity. (**b**) Production and characterization of recombination proteins**.** The recombinant proteins were purified and confirmed by SDS-PAGE and Western blot using anti-tBspA and anti-FlaB antibodies raised in mice. (**c**) NF-κB reporter assay. The TLR5-dependent NF-κB stimulating activity was measured to select an appropriate fusion protein among eight fusion variants: FlaB-tBspA (B-tBspA), FlaB-LK1-tBspA (B-L1-tB), FlaB-LK2-tBspA (B-L2-tB), FlaB-LK3- tBspA (B-L3-tB), tBspA-FlaB (tB-B), tBspA-LK1-FlaB (tB-L1-B), tBspA-LK2-FlaB (tB-L2-B), and tBspA-LK3-FlaB (tB-L3-B). **(d)** Determination of tBspA-specific antibody production. Serum and saliva samples were collected 2 weeks after the final immunization to measure tBspA-specific antibody responses using ELISA. The results are presented as mean ± SEM for each group. $n = 5$ $^{**}P < 0.01$

Supplementary Figure S4. Representative immunohistochemical staining of MPO in gingival tissue sections using confocal microscopy. (**a**) Immunization and experimental schedule. (**b**) Myeloperoxidase staining was performed at 8 days (day 15) after the final infection. MPO is visualized in red (Alexa Fluor 555), and Hoechst nuclear staining is shown in blue. The merged channel demonstrates the co-localization of MPO and nuclear staining. The image captures MPO-

infiltrating cells at the ligature site between the first and second molars, shown at 200X magnification by confocal microscopy.

Supplementary Figure S5. Optimization of the timing of periodontal inflammatory cytokine measurements**.** **(a)** Experimental schedule of periodontal inflammation within a temporary ligature plus oral infection model in BALB/c mice. Ligature was placed on day 0, followed by oral bacterial infection of mixed *Tf*, *Pg*, and *Fn*. The ligature was removed a day later, followed by another round of oral bacterial infection. Subgingival tissues were collected for mRNA gene expression analysis at the indicated time points 12-, 24-, and 48-hours post-ligature removal or post-final oral bacterial infection. (**b**) Quantitative RT-PCR analysis of IL-1β, IL-6, TNF-α, and MMP9 in mouse gingival tissues at various time points. Naïve, control mice; Lig, mice with ligature placement; Lig+OI, mice with ligature plus oral infection; LigR, mice with ligature placement/ removal; LigR+OI, mice with ligature placement/removal plus mixed oral bacterial infections. The results are presented as the mean ± SEM for each group. $n = 3$ *$P < 0.05$, **$P < 0.01$, NS non-significance.

Supplementary Figure S6. Detection of cognate antigens expressed on the surface of live bacteria *Tf*, *Pg*, and *Fn*. **(a-c)** Immunofluorescence detection of the natural form of BspA, Hgp44, and FomA expressing on the surface of live *Tf* , *Pg*, and *Fn*, respectively. Freshly cultured bacteria were incubated with antisera obtained from corresponding vaccines. The specimens were visualized using confocal microscopy.

Supplementary Figure S7. Inhibition of *Tf* invasion into KB cells by antisera or anti-saliva induced by monovalent (BtB) or divalent vaccines (BtB + HB or BtB + BtA). The adhesion/invasion of *Tf* into KB cells was determined by using flow cytometry and confocal microscopy. CFSE-labelled *Tf* was pre-incubated with IgG purified from anti-sera (equivalent to 19 μl/well) or anti-saliva (equivalent to 1/4) obtained from mice immunized with a monovalent (anti-BtB) or divalent vaccines (anti-HtB + HB or anti-BtB + BtA) for 1 hour. Then, KB cells were infected with CFSE-labelled *Tf* at an MOI of 1:100 for 4 hours. (**a, c)** Cells were analyzed by flow cytometry after quenching the fluorescence of bacteria bound on the surface with trypan blue. (**b, d)** Representative confocal microscopic images illustrating the adhesion and invasion of *Tf* treated with purified IgG from anti-sera (equivalent to 19 μl/well) or anti-saliva (equivalent to 1/4 dilution), respectively. Data are represented as the mean ± SEM from three independent flow cytometry experiments. **$P < 0.01$, *** $P < 0.001$, **** $P < 0.0001$.

Supplementary Figure S8. Determination of *Fn* colonization in mice oral cavity within a temporary ligature plus oral infection model by qPCR. (**a)** Immunization and experimental schedule. BALB/c were intranasally immunized with PBS or trivalent vaccine (BtB + HB + BtA) three times at 2-week intervals. Two weeks after the final immunization, mice were orally infected with a mixture of *Tf*, *Pg*, and *Fn* (5 times), corresponding to the temporary ligature plus oral infection model. Oral swabs were collected at 2 days after the final infection. (**b)** Detection of *Fn* colonization in mice oral cavity by qPCR. Oral swabs were obtained from control mice (Naïve), mice immunized with PBS before ligature placement/removal plus mixed oral bacterial infections (LigR+OI), and mice vaccinated with BtB + HB + BtA before ligature placement/removal plus mixed oral bacterial infections (Vax+LigR+OI). There were 4 mice in each group. qPCR analysis was used to quantify the copy number of *Fn* 16S rRNA gene by referencing the standard curve.

Supplementary Table S1. Oligonucleotide primers were used for qPCR in this study.

Gene	Direction	Sequence
IL-1β	FORWARD	5' GAGCGCTCACGAACAGTTG 3'
	REVERSE	3' TTGGTTAAATGACCTGCAACA 5'
IL-6	FORWARD	5' CCGGAGAGGAGACTTCACAG 3'
	REVERSE	3' CAGAATTGC CATTGCACAAC 5'
TNF-α	FORWARD	5' GCTGAGCTCAAACCCTGGTA 3'
	REVERSE	3' CGGACTCCGCAAAGTCTAAG 5'
MMP-9	FORWARD	5' ACGACATAGACGGCATCCA 3'
	REVERSE	3' GCTGTGGTTCAGTTGTGGTG 5'
MMP-3	FORWARD	5' TGGACGGCTAATGCTGGT 3'
	REVERSE	3' TTGGCTGAGTGGTAGAGTCCC 5'
CXCL-2	FORWARD	5' TCCAGAGCTTGAGTGTGACG 3'
	REVERSE	3' TCCAGGTCAGTTAGCCTTGC 5'
RANKL	FORWARD	5' GCA AGCTTG AAGCTCAGCCT 3'
	REVERSE	3' CCTCTCCAGACCGTAACTTA 5'
L32	FORWARD	5' GAGCTGCTACAACGGCAAC 3'
	REVERSE	3' TGGACGGCTAATGCTGGT 5'

Supplementary Table S2. Bacterial strains and plasmids were used in this study.

Bacteria	Description	Source
T. forsythia ATCC 430377		America Type Culture Collection
P. gingivalis ATCC 33277		[1]
F. nucleatum ATCC 10953		[1]
E. coli DH5*α*	F$^-$ *recA1* restriction negative	Laboratory collection
E. coli ER2566	F$^-$ *λ*$^-$ *fhuA2 [lon] ompT lacZ::T7 gene1galsulA11Δ(mcrCmrr)114::IS10R(mcr73::miniTn10TetS)2R(zgb210::Tn10)(* TetS*)endA1 [dcm]*	New England Biolabs, Inc.
E. coli BL 21 (DE3)	*hsdS gal (λcIts857 ind1 Sam7 nin5 lacUV5-T7 gene1)*	Laboratory collection
**Plasmids**		
pTYB12	N-terminal fusion expression vector in which the N terminus of a target protein is a fused Intein-tag; Ap^r	New England Biolabs, Inc.
pET30a(+)	N-terminal fusion expression vector in which the N terminus of a target protein is a fused His-tag; Km^r	EMD Bioscience
pET30a(+)::tBspA	596-bp *NdeI-XhoI* fragment containing ORF of *BspA* encoding (amino acid 883-1081) *T. forsythia* ATCC 43037 cloned into pET30a(+) plasmid	This study
pET30a(+)::B-tB	pET30a(+) plasmid containing a DNA-fragment of *flaB* fused with *tBspA* without linker (*NdeI-SalI-XhoI*)	This study
pET30a(+)::B-L1-tB	pET30a(+) plasmid containing a DNA-fragment of *flaB* fused with linker 1 and *tBspA* (*NdeI-SalI-NotI-XhoI*)	This study
pET30a(+)::B-L2-tB(BtB)	pET30a(+) plasmid containing a DNA-fragment of *flaB* fused with linker 2 and *tBspA* (*NdeI-SalI-NotI-XhoI*)	This study
pET30a(+)::B-L3-tB	pET30a(+) plasmid containing a DNA-fragment of *flaB* fused with linker 3 and *tBspA* (*NdeI-SalI-NotI-Xho*I)	This study
pET30a(+)::tB-B	pET30a(+) plasmid containing a DNA-fragment of *tBpA* fused with *flaB* without linker (*NdeI-SalI - XhoI*)	This study
pET30a(+)::tB-L1-B	pET30a(+) plasmid containing a DNA-fragment of *tBpA* fused with linker 1 and *flaB* (*NdeI-SalI-NotI-XhoI*)	This study
pET30a(+)::tB-L2-B	pET30a(+) plasmid containing a DNA-fragment of *tBpA* fused with linker 2 and *flaB* (*NdeI-SalI-NotI-XhoI*)	This study

pET30a(+)::tB-L3-B	pET30a(+) plasmid containing a DNA-fragment of *tBpA* fused with linker 3 and *flaB* (*NdeI-SalI-NotI-XhoI*)	This study
pTYB12::*flaB*	1.5-kb *EcoRI-PstI* fragment containing ORF of *flaB* cloned into pTYB12	[2]
pTYB12::H-LK3-B (HB)	pTYB12 plasmid containing a DNA-fragment of *hgp44* fused with linker 3 and *flaB* (*NdeI-S*alI-*NotI- EcoRI*)	[1]
pET30a(+)::B-LK2-tA (BtA)	pET30a(+) plasmid containing a DNA-fragment of *flaB* fused with linker2 and *tfomA* (*NdeI-SalI-NotI-XhoI*)	[1]

Linker1 (L1) 12-amino acid, *Linker2* (L2) 24-amino acid, *Linker3* (L3) 36 amino acid

Materials and Methods for Supplementary Figures

Determination of the optimal antigen of *Tf* by computational analysis

The virulence factor BspA from *Tf* 43037 was retrieved from the NCBI GenBank server (https://www.ncbi.nlm.nih.gov/nuccore/AF054892.1) under the accession number AF054892.1 with 1081 amino acids. The IEDB Server (tools.iedb.org/bcell/) demonstrated the B-cell epitopes (BepiPred2.0) and immunogenicity. Utilizing computational analysis, we identified truncated BspA (tBspA) as an optimal vaccine antigen for *Tf*. The selection process involved BepiPred 2.0 for B cell epitopes and Tongaokar antigenicity [3].

Plasmid construction and protein purification

To produce the recombinant tBspA protein, the tBspA DNA sequence, located at the C-terminal of the BspA domain outside the Leucine-rich repeat (LRR) region from the genomic DNA of *Tf* and corresponding to amino acid residues 883 to 1081 was amplified by PCR using primers set: forward primer 5′ GGGAATTC CATATG GCCGAACAGATGGTTTCCGT3 ′ and reverse primer 5′ CCG CTCGAG CTTTATAAGAATTTTGGTTACCCG 3′. The underlined sequences are endonuclease restriction sites necessary for subsequent cloning into vectors. The amplified DNA fragment was cloned into pET30a+ plasmid (Novagen, 69909-Merch Millipore). To enhance the improved purification of a recombinant fusion protein, linker peptides sourced from PspA of *Streptococcus pneumoniae* [4] were introduced between the FlaB component and the tBspA antigen (Supplementary Table S2). To produce FlaB-tBspA fusion proteins (B-tB, B-L1-tB, B-L2-tB, and B-L3-tB), the tBspA specific DNA fragment was amplified by PCR using primers set: forward primer 5′ AAATAT GCGGCCGC GCCGAACAGATGGTTTCCGT 3′ and reverse primer 5′ CCG CTCGAG CTTTATAAGAATTTTGGTTACCCG 3′ with *NotI-XhoI* overhangs and cloned at the C-terminus of the FlaB-linker peptides (L1, L2, and L3) specific DNA sequence which containing *NdeI-NotI* overhangs in pET30a+ vector. Similarly, to produce tBpA-FlaB fusion proteins (tB-B, tB-L1-B, tB-L2-B, and tB-L3-B), the tBspA specific DNA fragment was amplified by PCR using primers set: forward primer 5′ GGGAATTC CATATG GCCGAACAGATGGTTTCCGT 3′ and reverse primer 5′ ACGC GTCGAC CTTTATAAGAATTTTGGTTACCCG 3′ with *NdeI-SalI* overhangs and cloned at N-terminus of L1-, L2-, L3-FlaB specific DNA sequence having *SalI-XhoI* overhang in pET30a+ vector. The DNA sequences of the resulting expression vectors were confirmed by the dideoxy-chain termination sequencing method via the Macrogen Online Sequencing Order System (http://dna.macrogen.com/kor/).

The resultant expression plasmids were transformed into competent *E. coli* BL21. Protein expression was induced with 0.1 mM isopropyl-β-D-thiogalactoside (IPTG) for 18 h incubation at 18 °C, and cells were collected by centrifugation, then stored at −80 °C until needed. The bacterial pellets from 1 L culture were lysed using a 50 mL buffer (pH 8, 50 mM NaH_2PO_4, 300 mM NaCl, 10 mM imidazole, 0.1% TritonX-100, 0.1% Tween, and 20 μM phenylmethylsulfonyl fluoride) accompanying with sonication for 15 min at 5 s ON and 5 s OFF cycles. The experiments were done on ice. Following centrifugation at 18,000 rpm for 30 min, the resulting cell-free supernatant was loaded onto a column filled with Ni-NTA agarose beads (Qiagen, Hilden, Germany) following the manufacturer's instructions. The wild-type recombinant FlaB protein generated from pTYB12::*flaB* plasmid and protein purification was carried out as previously protocol [2]. Recombinant protein purity was verified through sodium dodecyl sulfate-polyacrylamide gel electrophoresis (SDS-PAGE), subsequent Western blot analysis using anti-FlaB and anti-tBspA antibodies raised in mice with complete Freund's adjuvant (Sigma-Aldrich, St. Louis, MO). The buffer for column-purified proteins was exchanged into phosphate-buffered saline (PBS) using centrifugal filter tubes (Amicon® Ultra-15 Centrifugal Filter, 10k). Lipopolysaccharide (LPS) contamination was removed by treatment with TritonX-114 (Sigma-Aldrich, St. Louis, MO, USA), and any residual Triton X-114 was removed by incubating with Bio-Beads™ SM-2 (Bio-Rad Laboratories, Inc., Hercules, CA, USA), following the manufacturer's instructions, using 0.3 g of Bio-beadsTM-2 per 1 ml of protein. Residual LPS content was determined using the gel-clotting Endosafe LAL kit (Charles River, Charleston, SC). The LPS levels in protein preparations

complied with Food and Drug Administration (FDA) guidelines (less than 0.15 EU/30 g per mouse).

Western blotting analysis

The recombinant proteins were separated using SDS-PAGE gels and transferred to nitrocellulose membranes (Amersham, Marlborough, MA, USA, 10600004). The anti-tBspA and anti-FlaB were diluted in PBST. These diluted sera were then applied to the respective membranes and incubated for 2 hours at room temperature (RT) to detect corresponding proteins. A horseradish peroxidase (HRP)-conjugated secondary antibody (Dako, Santa, CA, USA, P0260) was used for protein visualization. The visualization process was performed following the manufacturer's instructions.

NF-κB luciferase reporter assay

We evaluated the TLR5-stimulating activities of FlaB-fusion proteins by determining their TLR5-dependent NF-κB stimulating activity following procedures described in a previous study [1]. The TLR5-dependent NF-κB transfected HEK293T cells were treated with LPS-free recombinant proteins following equivalent molar ratios (100 ng FlaB, 152.52 ng B-tB or tB-B, 155.70 ng B-L1-tB or tB-L1-B, 158.88 ng B-L2-tB or tB-L2-B, and 162.07 ng B-L3-tB or tB-L3-B) for 18 hours. The PBS-treated cells were the control for the experiment. The cells were then lysed using a lysis buffer (Promega, E153A), luciferase activity was quantified using a luminometer (MicroLumat-Plus LB 96V, Berthold, Wilbad, Germany), and the fold-change activity was calculated by referencing the negative control.

Immunohistochemical staining of MPO in gingival tissue sections

A sliced 5 mm thick section was deparaffinized in xylene and hydrated by moving through a gradient of ethanol concentration through a gradient of ethanol concentrations 100%, 95%, 80%, 70%, and 50% ethanol), followed by rinsing with distilled water. Immunofluorescence (IF) staining for anti-MPO was carried out by prescribed methods [5]. The antigen is retrieved by heating the section at 98oC in citrate buffer (pH = 6.0) for 10 minutes, then cooling it at room temperature. Subsequently, the slide was washed twice with PBS. The sections were locked with 5% BSA in PBST (PBS + 0.5% Tween 20) to block non-specific binding for 1 hour. Next, the sections were stained with primary anti-MPO antibody (R&D, MI, USA, AF3667) at a 1:500 dilution in PBST at 4°C overnight. Afterward, the sections were rinsed with PBST 2 times before staining secondary antibody Alexa flour conjugated secondary antibody 555 (Invitrogen, Eugene, OR, USA, A-21422) at a 1:500 dilution in PBST for 1 hour at RT. The sections were stained with Hoechst 33342 for 15 minutes at room temperature to visualize the nucleus. Image confocal was performed using an LSM510 confocal fluorescence microscope.

Detection of *Fn* colonization in mouse oral cavity

Seven-week-old female BALB/c (*n* =4) were intranasally immunized with PBS or a trivalent vaccine (BtB + HB + BtA) 3 times at 2-week intervals. Two weeks after the third immunization, mice underwent ligature placement/removal plus oral infection with a mixture of *Tf*, *Pg*, and *Fn* (1 x 10^9 CFU of each in 100 μl/mouse) five times. Oral swabs were collected 2 days after the final infection by scraping the entire mouse dental area using 2 micro cotton swabs. Swabs were obtained from control mice (Naïve), mice with ligature placement/removal plus oral bacterial infection (LigR+OI), or mice vaccinated with BtB + HB + BtA prior ligature placement/removal plus oral bacterial infection (Vax+LigR+OI). Cotton swabs were immersed in 30 μl of PBS, and 3 μl out of the 30 μl were used as a template for detecting *Fn* through the qPCR, which targeted the 16S rRNA gene of *Fn* using SYBR Green qPCR PreMix (Enzynomic, Daejeon, South Korea, RT501M). The colony forming unit (CFU) of *Fn* was quantified by referencing the standard curve.

References

1. Puth, S.; Hong, S.H.; Na, H.S.; Lee, H.H.; Lee, Y.S.; Kim, S.Y.; Tan, W.; Hwang, H.S.; Sivasamy, S.; Jeong, K.; et al. A built-in adjuvant-engineered mucosal vaccine against dysbiotic periodontal diseases. *Mucosal Immunology* **2019**, *12*, 565-579, doi:10.1038/S41385-018-0104-6.

2. Lee, S.E.; Kim, S.Y.; Jeong, B.C.; Kim, Y.R.; Bae, S.J.; Ahn, O.S.; Lee, J.J.; Song, H.C.; Kim, J.M.; Choy, H.E.; et al. A bacterial flagellin, Vibrio vulnificus FlaB, has a strong mucosal adjuvant activity to induce protective immunity. *Infection and Immunity* **2006**, *74*, 694-702, doi:10.1128/IAI.74.1.694-702.2006.

3. Jespersen, M.C.; Peters, B.; Nielsen, M.; Marcatili, P. BepiPred-2.0: improving sequence-based B-cell epitope prediction using conformational epitopes. *Nucleic acids research* **2017**, *45*, W24-W29.

4. Nguyen, C.T.; Kim, S.Y.; Kim, M.S.; Lee, S.E.; Rhee, J.H. Intranasal immunization with recombinant PspA fused with a flagellin enhances cross-protective immunity against Streptococcus pneumoniae infection in mice. *Vaccine* **2011**, *29*, 5731-5739, doi:10.1016/J.VACCINE.2011.05.095.

5. Pierre, J.; Hinterleitner, R.; Bouziat, R.; Hubert, N.; Leone, V.; Miyoshi, J.; Jabri, B.; Chang, E. Data on changes to mucosal inflammation and the intestinal microbiota following dietary micronutrients in genetically susceptible hosts. *Data in brief* **2018**, *20*, 387-393. doi: 10.1016/j.dib.2018.08.026
